# 2-{[2,8-Bis(trifluoro­meth­yl)quinolin-4-yl](hy­droxy)meth­yl}piperidin-1-ium 3-amino-5-nitro­benzoate sesquihydrate

**DOI:** 10.1107/S160053681104270X

**Published:** 2011-10-22

**Authors:** Marcus V. N. de Souza, James L. Wardell, Solange M. S. V. Wardell, Seik Weng Ng, Edward R. T. Tiekink

**Affiliations:** a Fundação Oswaldo Cruz, Instituto de Tecnologia em Fármacos – Farmanguinhos, R. Sizenando Nabuco 100, Manguinhos, 21041-250 Rio de Janeiro, RJ, Brazil; b Centro de Desenvolvimento Tecnológico em Saúde (CDTS), Fundação Oswaldo Cruz (FIOCRUZ), Casa Amarela, Campus de Manguinhos, Av. Brasil 4365, 21040-900 Rio de Janeiro, RJ, Brazil; c CHEMSOL, 1 Harcourt Road, Aberdeen AB15 5NY, Scotland; d Department of Chemistry, University of Malaya, 50603 Kuala Lumpur, Malaysia; e Chemistry Department, Faculty of Science, King Abdulaziz University, PO Box 80203 Jeddah, Saudi Arabia

## Abstract

The asymmetric unit of the title salt solvate, C_17_H_17_F_6_N_2_O^+^·C_7_H_5_N_2_O_4_^−^·1.5H_2_O, comprises a piperidin-1-ium cation, a 3-amino-5-nitro­benzoate anion, and three fractionally occupied [*i.e*. 0.414 (3), 0.627 (6) and 0.459 (5)] disordered water mol­ecules of solvation. The cation has an L shape with a C—C—C—C torsion angle of −102.9 (3)° for the atoms linking the quinolinyl group to the rest of the cation. In the anion, the carboxyl­ate and nitro groups are essentially coplanar with the benzene ring [O—C—C—C torsion angle = 179.7 (2)° and O—N—C—C torsion angle = −3.9 (3)°]. In the crystal, extensive O—H⋯O, O—H⋯F and N—H⋯·O hydrogen bonding leads to the formation of a layer in the *ab* plane.

## Related literature

For background information on mefloquine and derivatives, see: Kunin & Ellis (2007 ▶); Maguire *et al.* (2006 ▶); Dow *et al.* (2004 ▶). For selected crystal structures of mefloquine and its salts, see: Obaleye *et al.* (2009 ▶); Skórska *et al.* (2005 ▶); Karle & Karle (1991 ▶, 2002 ▶); Wardell *et al.* (2010 ▶, 2011 ▶).
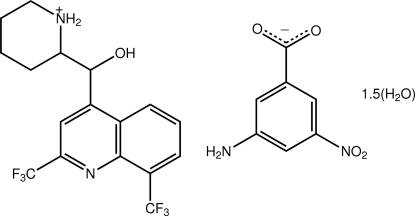


## Experimental

### 

#### Crystal data

C_17_H_17_F_6_N_2_O^+^·C_7_H_5_N_2_O_4_^−^·1.5H_2_O
*M* *_r_* = 587.48
Triclinic, 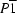

*a* = 9.1705 (5) Å
*b* = 12.5446 (9) Å
*c* = 12.7788 (8) Å
α = 66.278 (4)°
β = 77.261 (4)°
γ = 71.537 (4)°
*V* = 1269.23 (14) Å^3^
*Z* = 2
Mo *K*α radiation
μ = 0.14 mm^−1^
*T* = 120 K
0.28 × 0.16 × 0.10 mm

#### Data collection

Bruker–Nonius APEXII CCD camera on κ-goniostat diffractometer
Absorption correction: multi-scan (*SADABS*; Sheldrick, 2007 ▶) *T*_min_ = 0.640, *T*_max_ = 0.746
25681 measured reflections
5832 independent reflections
3477 reflections with *I* > 2σ(*I*)
*R*_int_ = 0.078

#### Refinement

*R*[*F* ^2^ > 2σ(*F* ^2^)] = 0.064
*wR*(*F* ^2^) = 0.174
*S* = 1.03
5832 reflections
416 parameters
15 restraints
H atoms treated by a mixture of independent and constrained refinement
Δρ_max_ = 0.39 e Å^−3^
Δρ_min_ = −0.38 e Å^−3^

### 

Data collection: *COLLECT* (Hooft, 1998 ▶); cell refinement: *DENZO* (Otwinowski & Minor, 1997 ▶) and *COLLECT*; data reduction: *DENZO* and *COLLECT*; program(s) used to solve structure: *SHELXS97* (Sheldrick, 2008 ▶); program(s) used to refine structure: *SHELXL97* (Sheldrick, 2008 ▶); molecular graphics: *ORTEP-3* (Farrugia, 1997 ▶) and *DIAMOND* (Brandenburg, 2006 ▶); software used to prepare material for publication: *publCIF* (Westrip, 2010 ▶).

## Supplementary Material

Crystal structure: contains datablock(s) global, I. DOI: 10.1107/S160053681104270X/lh5355sup1.cif

Structure factors: contains datablock(s) I. DOI: 10.1107/S160053681104270X/lh5355Isup2.hkl

Additional supplementary materials:  crystallographic information; 3D view; checkCIF report

## Figures and Tables

**Table 1:** Hydrogen-bond geometry (Å, °)

*D*—H⋯*A*	*D*—H	H⋯*A*	*D*⋯*A*	*D*—H⋯*A*
O1—H1⋯O2	0.84 (1)	1.83 (1)	2.669 (3)	178 (3)
N4—H41⋯O2^i^	0.88 (1)	2.04 (1)	2.900 (3)	164 (3)
N2—H21⋯O3^ii^	0.89 (1)	1.87 (1)	2.717 (3)	159 (3)
N4—H42⋯O5^iii^	0.88 (1)	2.27 (2)	3.101 (3)	159 (3)
N2—H22⋯O2*W*	0.89 (1)	2.05 (1)	2.916 (4)	165 (3)
N2—H22⋯O3*W*	0.89 (1)	1.98 (2)	2.727 (5)	141 (3)
OWw—H1*W*1⋯O1	0.84 (1)	2.08 (4)	2.867 (5)	156 (8)
O1*W*—H1*W*2⋯F6^iv^	0.84 (1)	2.47 (7)	2.867 (5)	110 (6)
O2*W*—H2*W*1⋯O1*W*	0.85 (1)	2.07 (2)	2.859 (6)	154 (4)
O2*W*—H2*W*2⋯O4^v^	0.85 (1)	2.30 (2)	3.119 (4)	165 (4)
O3*W*—H3*W*1⋯O3	0.84 (1)	2.21 (1)	3.049 (5)	174 (7)
O3*W*—H3*W*2⋯O4^v^	0.84 (1)	2.37 (3)	3.158 (5)	156 (7)
O3*W*—H3*W*2⋯O5^v^	0.84 (1)	2.32 (6)	3.016 (5)	140 (7)

Symmetry codes: (i) 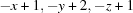; (ii) 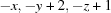; (iii) 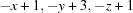; (iv) 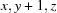; (v) 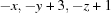.

**Fig. 1.**
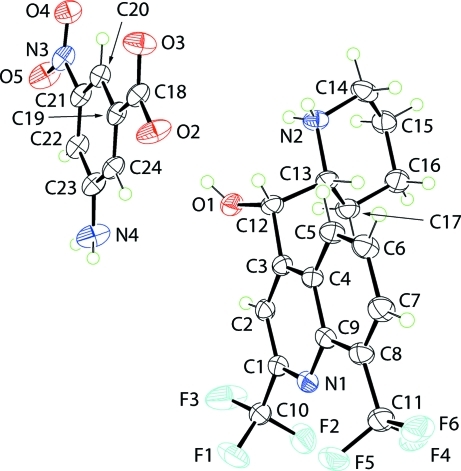
The molecular structures of the components comprising the asymmetric unit in (I) showing the atom-labelling scheme and displacement ellipsoids at the 50% probability level. The disordered water molecules of solvation have been omitted for reasons of clarity.

**Fig. 2.**
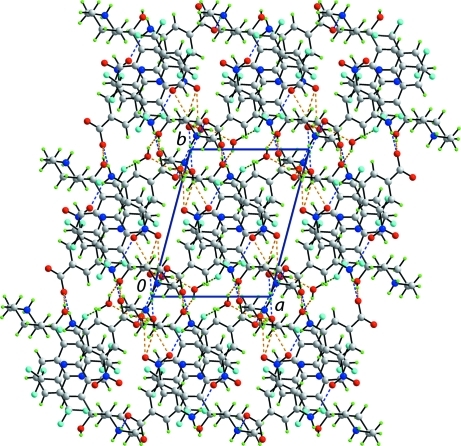
A view in projection along the a axis of the unit-cell contents in (I) showing the alternation of cations and anions along the c axis. The O—H···O, N—H···O and O—H···F interactions are shown as orange, blue and green dashed lines, respectively.

## supplementary crystallographic information

### Comment

A synthetic analogue of quinine, mefloquine, is manufactured as the racemic *erythro* hydrochloride salt, and is used in the prevention and treatment of malaria in combination with other drugs (Maguire *et al.*, 2006). While it is known that both enantiomers of *erythro* mefloquinium hydrochloride are active, the (+) form is the more potent against the D6 and W2 strains of *Plasmodium falciparum* (Karle & Karle, 2002). After its introduction in 1971, mefloquine has proved to be an effective anti-malarial agent, in part due to its long half-life and the fact that it is a good prophylactic. A widespread resistance of *Plasmodium sp*. developed by the end of the 20th century. This, together with undesirable side-effects [*e.g*. anxiety, aggression, seizures, nightmares, neuropathy, insomnia, acute depression and urinary disorders] have resulted in a decline in its use (Dow *et al.*, 2004). Derivatives of mefloquine are also been investigated against for efficacy against other diseases, *e.g*. as anti-viral and anti-tuberculosis agents (Kunin & Ellis, 2007). A few crystal structures of mefloquine (Skórska *et al.*, 2005) and mefloquinium salts, including the hydrated chloride (Karle & Karle, 2002; Skórska *et al.*, 2005), methylsulfonate (Karle & Karle, 1991), tetrachlorocobaltate (Skórska *et al.*, 2005), and tetrachlorocuprate and tetrabromocadmate salts (Obaleye *et al.*, 2009) have been reported. We now report the structure of the title salt, (I), in continuation of structural studies in this area (Wardell *et al.*, 2010; Wardell *et al.*, 2011).

The asymmetric unit of (I) comprises a piperidin-1-ium cation, a 3-amino-5-nitrobenzoate anion, and three fractionally occupied [*i.e*. 0.414 (3), 0.627 (6) and 0.459 (5)] disordered water molecules of solvation; the ions are illustrated in Fig. 1. The confirmation of protonation at the amine-N2 atom is found in the nature of the intermolecular interactions, see below, as well as in the equivalence of the C—O bond distances [O2—C18 = 1.251 (4) Å and O3—C18 = 1.246 (4) Å] in the anion. Overall, the cation has an *L*-shaped conformation as the quinolinyl residue is approximately orthogonal to the rest of the cation; the C2–C3–C12–C13 torsion angle is -102.9 (3) °. The six-membered piperidin-1-ium ring adopts a chair conformation. The anion is effectively planar with both of the carboxylate [O2—C18—C19—C20 torsion angle = 179.7 (2)°] and nitro [O4—N3—C21—C20 torsion angle = -3.9 (3)°] groups being co-planar with the benzene ring to which it is connected

The crystal packing is stabilized by hydrogen bonding, Table 1, that leads to layers in the *ab* plane. The carboxylate-O2 atom accepts two hydrogen bonds, one from the cation-OH and the other from the anion-amino-H. The carboxylate-O3 atom accepts a single interaction, *i.e*. from an ammonium-H. The second amino-H atom connects to nitro-O while the second ammonium-H hydrogen bonds water-O atoms. Water molecules form hydrogen bonds with each other and also donor hydrogen bonds to the cation-O atom, anion-O atoms and a weak contact to the F6 atom, Table 1. A view of the unit-cell contents is shown in Fig. 2 indicating layers stack along the *c* axis.

### Experimental

A solution of mefloquine (0.383 g, 1 mmol) and 3-amino-5-nitrobenzoic acid (0.18 g. 1 mmol) in EtOH (15 ml) was refluxed for 20 min. On maintaining the reaction mixture at room temperature, crystals of the title salt slowly formed. *M*.pt.: 503–504 K (dec.). IR ν: 3600–2200(br), 1632, 1612, 1555, 1518, 1472, 1435, 1389, 1344, 1310, 1265, 1217, 1184, 1150, 1109, 1078, 1013, 970, 934, 918, 899, 887, 866, 839, 795, 783, 743, 725, 665, 542, 444 cm^-1^.

### Refinement

The C-bound H atoms were geometrically placed (C—H = 0.95–1.00 Å) and refined as riding with *U_iso_*(H) = 1.2*U_eq_*(C). The O—H and N—H H atoms were located from a difference map and refined with O—H = 0.84±0.01 Å and N—H = 0.88–0.92±0.01 Å, respectively, and with *U_iso_*(H) = *yU_eq_*(O or N) with *y* = 1.5 for O and *y* = 1.2 for N. There are a total of 1.5 water molecules in the asymmetric unit and these are disordered over three positions with site occupancies factors of 0.414 (3), 0.627 (6) and 0.459 (5); hydrogen atoms were located for each of these and refined as described above.

### Crystal data

**Table:**

C_17_H_17_F_6_N_2_O^+^·C_7_H_5_N_2_O_4_^−^·1.5H_2_O	*Z* = 2
*M**_r_* = 587.48	*F*(000) = 606
Triclinic, *P*1	*D*_x_ = 1.537 Mg m^−^^3^
Hall symbol: -P 1	Mo *K*α radiation, λ = 0.71073 Å
*a* = 9.1705 (5) Å	Cell parameters from 30639 reflections
*b* = 12.5446 (9) Å	θ = 2.9–27.5°
*c* = 12.7788 (8) Å	µ = 0.14 mm^−^^1^
α = 66.278 (4)°	*T* = 120 K
β = 77.261 (4)°	Block, orange
γ = 71.537 (4)°	0.28 × 0.16 × 0.10 mm
*V* = 1269.23 (14) Å^3^	

### Data collection

**Table:**

Bruker–Nonius APEXII CCD camera on κ-goniostat diffractometer	5832 independent reflections
Radiation source: Bruker-Nonius FR591 rotating anode	3477 reflections with *I* > 2σ(*I*)
10cm confocal mirrors	*R*_int_ = 0.078
Detector resolution: 9.091 pixels mm^-1^	θ_max_ = 27.6°, θ_min_ = 3.0°
φ & ω scans	*h* = −11→11
Absorption correction: multi-scan (*SADABS*; Sheldrick, 2007)	*k* = −16→16
*T*_min_ = 0.640, *T*_max_ = 0.746	*l* = −16→16
25681 measured reflections	

### Refinement

**Table:**

Refinement on *F*^2^	Secondary atom site location: difference Fourier map
Least-squares matrix: full	Hydrogen site location: inferred from neighbouring sites
*R*[*F*^2^ > 2σ(*F*^2^)] = 0.064	H atoms treated by a mixture of independent and constrained refinement
*wR*(*F*^2^) = 0.174	*w* = 1/[σ^2^(*F*_o_^2^) + (0.0836*P*)^2^ + 0.2367*P*] where *P* = (*F*_o_^2^ + 2*F*_c_^2^)/3
*S* = 1.03	(Δ/σ)_max_ < 0.001
5832 reflections	Δρ_max_ = 0.39 e Å^−^^3^
416 parameters	Δρ_min_ = −0.38 e Å^−^^3^
15 restraints	Extinction correction: *SHELXL97*, Fc^*^=kFc[1+0.001xFc^2^λ^3^/sin(2θ)]^-1/4^
Primary atom site location: structure-invariant direct methods	Extinction coefficient: 0.032 (4)

### Special details

**Table:**

Geometry. All s.u.'s (except the s.u. in the dihedral angle between two l.s. planes) are estimated using the full covariance matrix. The cell s.u.'s are taken into account individually in the estimation of s.u.'s in distances, angles and torsion angles; correlations between s.u.'s in cell parameters are only used when they are defined by crystal symmetry. An approximate (isotropic) treatment of cell s.u.'s is used for estimating s.u.'s involving l.s. planes.
Refinement. Refinement of *F*^2^ against ALL reflections. The weighted *R*-factor *wR* and goodness of fit *S* are based on *F*^2^, conventional *R*-factors *R* are based on *F*, with *F* set to zero for negative *F*^2^. The threshold expression of *F*^2^ > 2σ(*F*^2^) is used only for calculating *R*-factors(gt) *etc*. and is not relevant to the choice of reflections for refinement. *R*-factors based on *F*^2^ are statistically about twice as large as those based on *F*, and *R*- factors based on ALL data will be even larger.

### Fractional atomic coordinates and isotropic or equivalent isotropic displacement parameters (Å^2^)

**Table:**

	*x*	*y*	*z*	*U*_iso_*/*U*_eq_	Occ. (<1)
F1	0.87381 (17)	0.49471 (18)	0.83650 (15)	0.0532 (5)	
F2	0.74544 (19)	0.50218 (18)	0.99348 (14)	0.0515 (5)	
F3	0.7703 (2)	0.66464 (17)	0.8535 (2)	0.0766 (7)	
F4	0.55227 (19)	0.21925 (14)	0.99220 (14)	0.0443 (4)	
F5	0.69944 (17)	0.22502 (14)	0.83406 (15)	0.0457 (5)	
F6	0.51655 (18)	0.13558 (14)	0.88618 (15)	0.0443 (4)	
O1	0.3030 (2)	0.89697 (16)	0.68621 (16)	0.0321 (4)	
H1	0.287 (4)	0.940 (2)	0.6179 (12)	0.048*	
O2	0.2506 (2)	1.03009 (17)	0.46810 (18)	0.0442 (5)	
O3	0.0666 (2)	1.19038 (17)	0.39087 (19)	0.0437 (5)	
O4	0.0825 (2)	1.58565 (17)	0.37663 (17)	0.0409 (5)	
O5	0.2815 (3)	1.60038 (18)	0.42819 (19)	0.0509 (6)	
O1W	0.3447 (6)	1.0482 (5)	0.7901 (4)	0.0431 (14)	0.414 (3)
H1W1	0.358 (8)	0.991 (5)	0.768 (7)	0.065*	0.414 (3)
H1W2	0.413 (7)	1.086 (6)	0.754 (6)	0.065*	0.414 (3)
O2W	0.0428 (4)	1.1348 (3)	0.7158 (3)	0.0449 (11)	0.627 (6)
H2W1	0.128 (3)	1.133 (4)	0.733 (5)	0.067*	0.627 (6)
H2W2	−0.006 (5)	1.2078 (17)	0.689 (5)	0.067*	0.627 (6)
O3W	−0.0625 (6)	1.1518 (4)	0.6409 (4)	0.0467 (16)	0.459 (5)
H3W1	−0.022 (9)	1.158 (6)	0.573 (3)	0.070*	0.459 (5)
H3W2	−0.096 (9)	1.221 (3)	0.644 (6)	0.070*	0.459 (5)
N1	0.5885 (2)	0.46024 (19)	0.84811 (18)	0.0284 (5)	
N2	−0.0203 (2)	0.9090 (2)	0.7489 (2)	0.0297 (5)	
H21	−0.039 (3)	0.895 (3)	0.6913 (17)	0.036*	
H22	0.008 (3)	0.9766 (16)	0.727 (2)	0.036*	
N3	0.2069 (3)	1.5407 (2)	0.41647 (19)	0.0338 (5)	
N4	0.6061 (3)	1.1835 (2)	0.5903 (2)	0.0467 (7)	
H41	0.657 (3)	1.1130 (16)	0.585 (3)	0.056*	
H42	0.660 (3)	1.230 (3)	0.590 (3)	0.056*	
C1	0.6030 (3)	0.5648 (2)	0.8371 (2)	0.0273 (6)	
C2	0.4964 (3)	0.6767 (2)	0.7926 (2)	0.0269 (6)	
H2A	0.5132	0.7488	0.7899	0.032*	
C3	0.3666 (3)	0.6788 (2)	0.7529 (2)	0.0261 (6)	
C4	0.3486 (3)	0.5686 (2)	0.7576 (2)	0.0262 (6)	
C5	0.2229 (3)	0.5601 (2)	0.7155 (2)	0.0295 (6)	
H5	0.1475	0.6313	0.6800	0.035*	
C6	0.2094 (3)	0.4515 (2)	0.7256 (2)	0.0320 (6)	
H6A	0.1254	0.4478	0.6962	0.038*	
C7	0.3189 (3)	0.3440 (2)	0.7793 (2)	0.0325 (6)	
H7	0.3071	0.2686	0.7868	0.039*	
C8	0.4418 (3)	0.3481 (2)	0.8204 (2)	0.0295 (6)	
C9	0.4616 (3)	0.4604 (2)	0.8081 (2)	0.0273 (6)	
C10	0.7471 (3)	0.5578 (2)	0.8802 (2)	0.0322 (6)	
C11	0.5530 (3)	0.2330 (2)	0.8820 (3)	0.0350 (6)	
C12	0.2441 (3)	0.7967 (2)	0.7111 (2)	0.0278 (6)	
H12A	0.2092	0.8029	0.6396	0.033*	
C13	0.1041 (3)	0.7996 (2)	0.8030 (2)	0.0281 (6)	
H13	0.0664	0.7264	0.8232	0.034*	
C14	−0.1666 (3)	0.9248 (3)	0.8275 (3)	0.0371 (7)	
H14A	−0.2144	0.8581	0.8453	0.045*	
H14B	−0.2404	1.0013	0.7885	0.045*	
C15	−0.1351 (3)	0.9265 (3)	0.9384 (2)	0.0374 (7)	
H15A	−0.2316	0.9299	0.9914	0.045*	
H15B	−0.1005	0.9994	0.9217	0.045*	
C16	−0.0107 (3)	0.8142 (3)	0.9959 (2)	0.0385 (7)	
H16A	0.0126	0.8192	1.0656	0.046*	
H16B	−0.0491	0.7416	1.0196	0.046*	
C17	0.1353 (3)	0.8042 (3)	0.9131 (2)	0.0328 (6)	
H17A	0.2141	0.7304	0.9506	0.039*	
H17B	0.1773	0.8742	0.8939	0.039*	
C18	0.1878 (3)	1.1404 (2)	0.4389 (2)	0.0297 (6)	
C19	0.2628 (3)	1.2185 (2)	0.4629 (2)	0.0273 (6)	
C20	0.1963 (3)	1.3416 (2)	0.4311 (2)	0.0275 (6)	
H20	0.1021	1.3781	0.3969	0.033*	
C21	0.2735 (3)	1.4088 (2)	0.4515 (2)	0.0294 (6)	
C22	0.4084 (3)	1.3611 (2)	0.5017 (2)	0.0322 (6)	
H22A	0.4565	1.4115	0.5137	0.039*	
C23	0.4745 (3)	1.2369 (2)	0.5350 (2)	0.0325 (6)	
C24	0.3990 (3)	1.1676 (2)	0.5141 (2)	0.0297 (6)	
H24	0.4425	1.0832	0.5356	0.036*	

### Atomic displacement parameters (Å^2^)

**Table:**

	*U*^11^	*U*^22^	*U*^33^	*U*^12^	*U*^13^	*U*^23^
F1	0.0231 (8)	0.0818 (14)	0.0522 (11)	−0.0005 (8)	−0.0032 (7)	−0.0318 (10)
F2	0.0431 (10)	0.0790 (14)	0.0330 (9)	−0.0209 (9)	−0.0030 (7)	−0.0173 (9)
F3	0.0609 (12)	0.0301 (11)	0.140 (2)	−0.0128 (9)	−0.0556 (13)	−0.0075 (12)
F4	0.0490 (10)	0.0316 (10)	0.0437 (10)	−0.0045 (7)	−0.0151 (8)	−0.0043 (8)
F5	0.0274 (8)	0.0313 (9)	0.0670 (12)	0.0030 (7)	−0.0015 (8)	−0.0161 (9)
F6	0.0432 (9)	0.0209 (9)	0.0658 (11)	−0.0029 (7)	−0.0133 (8)	−0.0129 (8)
O1	0.0339 (10)	0.0219 (10)	0.0374 (10)	−0.0080 (8)	−0.0063 (8)	−0.0055 (8)
O2	0.0627 (13)	0.0206 (11)	0.0550 (13)	−0.0047 (9)	−0.0330 (11)	−0.0102 (9)
O3	0.0380 (11)	0.0283 (11)	0.0703 (14)	−0.0028 (9)	−0.0203 (10)	−0.0201 (11)
O4	0.0492 (12)	0.0263 (11)	0.0478 (12)	−0.0050 (9)	−0.0130 (10)	−0.0133 (10)
O5	0.0781 (15)	0.0252 (11)	0.0601 (14)	−0.0162 (10)	−0.0286 (12)	−0.0129 (10)
O1W	0.050 (3)	0.044 (3)	0.040 (3)	−0.017 (2)	−0.003 (2)	−0.019 (3)
O2W	0.043 (2)	0.032 (2)	0.056 (2)	−0.0070 (15)	−0.0043 (17)	−0.0147 (18)
O3W	0.049 (3)	0.030 (3)	0.055 (3)	−0.005 (2)	−0.003 (2)	−0.014 (2)
N1	0.0231 (11)	0.0252 (12)	0.0322 (12)	−0.0050 (9)	0.0003 (9)	−0.0081 (10)
N2	0.0301 (11)	0.0209 (12)	0.0385 (13)	−0.0007 (9)	−0.0104 (10)	−0.0120 (10)
N3	0.0525 (15)	0.0229 (12)	0.0296 (12)	−0.0110 (11)	−0.0067 (11)	−0.0107 (10)
N4	0.0625 (17)	0.0285 (14)	0.0600 (17)	−0.0056 (12)	−0.0316 (14)	−0.0185 (13)
C1	0.0240 (12)	0.0243 (14)	0.0288 (13)	−0.0051 (10)	−0.0005 (10)	−0.0066 (11)
C2	0.0273 (13)	0.0208 (13)	0.0288 (13)	−0.0066 (10)	0.0010 (10)	−0.0069 (11)
C3	0.0259 (13)	0.0218 (13)	0.0250 (12)	−0.0032 (10)	−0.0005 (10)	−0.0060 (11)
C4	0.0239 (12)	0.0234 (14)	0.0273 (13)	−0.0038 (10)	−0.0009 (10)	−0.0077 (11)
C5	0.0267 (13)	0.0240 (14)	0.0338 (14)	0.0004 (10)	−0.0028 (11)	−0.0119 (12)
C6	0.0265 (13)	0.0310 (15)	0.0389 (15)	−0.0043 (11)	−0.0037 (11)	−0.0152 (13)
C7	0.0300 (14)	0.0257 (15)	0.0416 (15)	−0.0041 (11)	−0.0019 (12)	−0.0153 (13)
C8	0.0256 (13)	0.0246 (14)	0.0354 (14)	−0.0014 (10)	−0.0016 (11)	−0.0125 (12)
C9	0.0207 (12)	0.0269 (14)	0.0299 (13)	−0.0044 (10)	0.0010 (10)	−0.0089 (11)
C10	0.0311 (14)	0.0240 (14)	0.0366 (15)	−0.0069 (11)	−0.0026 (12)	−0.0065 (12)
C11	0.0308 (14)	0.0220 (14)	0.0465 (17)	−0.0028 (11)	−0.0034 (12)	−0.0099 (13)
C12	0.0268 (13)	0.0188 (13)	0.0336 (14)	−0.0023 (10)	−0.0065 (11)	−0.0061 (11)
C13	0.0261 (13)	0.0150 (13)	0.0379 (14)	−0.0004 (10)	−0.0057 (11)	−0.0067 (11)
C14	0.0246 (13)	0.0301 (16)	0.0555 (18)	−0.0004 (11)	−0.0057 (12)	−0.0186 (14)
C15	0.0319 (14)	0.0318 (16)	0.0440 (16)	−0.0014 (12)	0.0000 (12)	−0.0162 (14)
C16	0.0350 (15)	0.0324 (16)	0.0402 (16)	−0.0012 (12)	0.0003 (12)	−0.0131 (14)
C17	0.0294 (13)	0.0281 (15)	0.0371 (15)	−0.0019 (11)	−0.0072 (11)	−0.0102 (12)
C18	0.0413 (15)	0.0230 (14)	0.0255 (13)	−0.0078 (12)	−0.0068 (11)	−0.0081 (11)
C19	0.0386 (14)	0.0244 (14)	0.0201 (12)	−0.0106 (11)	−0.0022 (11)	−0.0077 (11)
C20	0.0354 (14)	0.0232 (14)	0.0258 (13)	−0.0089 (11)	−0.0023 (11)	−0.0102 (11)
C21	0.0457 (16)	0.0197 (13)	0.0235 (13)	−0.0080 (11)	−0.0043 (11)	−0.0083 (11)
C22	0.0469 (16)	0.0258 (15)	0.0300 (14)	−0.0127 (12)	−0.0069 (12)	−0.0121 (12)
C23	0.0470 (16)	0.0283 (15)	0.0272 (13)	−0.0104 (12)	−0.0108 (12)	−0.0106 (12)
C24	0.0422 (15)	0.0213 (14)	0.0254 (13)	−0.0069 (11)	−0.0070 (11)	−0.0073 (11)

### Geometric parameters (Å, °)

**Table:**

F1—C10	1.333 (3)	C4—C5	1.423 (4)
F2—C10	1.329 (3)	C4—C9	1.425 (4)
F3—C10	1.318 (3)	C5—C6	1.359 (4)
F4—C11	1.346 (3)	C5—H5	0.9500
F5—C11	1.338 (3)	C6—C7	1.414 (4)
F6—C11	1.345 (3)	C6—H6A	0.9500
O1—C12	1.417 (3)	C7—C8	1.367 (4)
O1—H1	0.839 (10)	C7—H7	0.9500
O2—C18	1.249 (3)	C8—C9	1.419 (4)
O3—C18	1.248 (3)	C8—C11	1.501 (4)
O4—N3	1.223 (3)	C12—C13	1.540 (3)
O5—N3	1.230 (3)	C12—H12A	1.0000
O1W—H1W1	0.843 (10)	C13—C17	1.523 (4)
O1W—H1W2	0.843 (10)	C13—H13	1.0000
O2W—H2W1	0.849 (10)	C14—C15	1.516 (4)
O2W—H2W2	0.845 (10)	C14—H14A	0.9900
O3W—H2W2	1.36 (5)	C14—H14B	0.9900
O3W—H3W1	0.843 (10)	C15—C16	1.532 (4)
O3W—H3W2	0.843 (10)	C15—H15A	0.9900
N1—C1	1.307 (3)	C15—H15B	0.9900
N1—C9	1.370 (3)	C16—C17	1.518 (4)
N2—C14	1.499 (3)	C16—H16A	0.9900
N2—C13	1.502 (3)	C16—H16B	0.9900
N2—H21	0.885 (10)	C17—H17A	0.9900
N2—H22	0.886 (10)	C17—H17B	0.9900
N3—C21	1.481 (3)	C18—C19	1.513 (4)
N4—C23	1.374 (4)	C19—C24	1.384 (4)
N4—H41	0.881 (10)	C19—C20	1.389 (4)
N4—H42	0.877 (10)	C20—C21	1.384 (3)
C1—C2	1.409 (4)	C20—H20	0.9500
C1—C10	1.506 (4)	C21—C22	1.368 (4)
C2—C3	1.382 (4)	C22—C23	1.399 (4)
C2—H2A	0.9500	C22—H22A	0.9500
C3—C4	1.420 (4)	C23—C24	1.399 (4)
C3—C12	1.520 (3)	C24—H24	0.9500
			
C12—O1—H1	105 (2)	O1—C12—C3	111.4 (2)
H1W1—O1W—H1W2	108.9 (18)	O1—C12—C13	108.89 (19)
H2W1—O2W—H2W2	107.6 (17)	C3—C12—C13	110.1 (2)
H2W2—O3W—H3W1	114 (6)	O1—C12—H12A	108.8
H2W2—O3W—H3W2	49 (6)	C3—C12—H12A	108.8
H3W1—O3W—H3W2	108.9 (18)	C13—C12—H12A	108.8
C1—N1—C9	116.9 (2)	N2—C13—C17	109.3 (2)
C14—N2—C13	113.7 (2)	N2—C13—C12	106.8 (2)
C14—N2—H21	108.8 (18)	C17—C13—C12	115.0 (2)
C13—N2—H21	103.5 (19)	N2—C13—H13	108.5
C14—N2—H22	105.2 (18)	C17—C13—H13	108.5
C13—N2—H22	112.4 (19)	C12—C13—H13	108.5
H21—N2—H22	113 (3)	N2—C14—C15	111.1 (2)
O4—N3—O5	122.9 (2)	N2—C14—H14A	109.4
O4—N3—C21	119.5 (2)	C15—C14—H14A	109.4
O5—N3—C21	117.6 (2)	N2—C14—H14B	109.4
C23—N4—H41	114 (2)	C15—C14—H14B	109.4
C23—N4—H42	118 (2)	H14A—C14—H14B	108.0
H41—N4—H42	117 (3)	C14—C15—C16	110.9 (2)
N1—C1—C2	125.9 (2)	C14—C15—H15A	109.5
N1—C1—C10	114.0 (2)	C16—C15—H15A	109.5
C2—C1—C10	120.1 (2)	C14—C15—H15B	109.5
C3—C2—C1	118.1 (2)	C16—C15—H15B	109.5
C3—C2—H2A	121.0	H15A—C15—H15B	108.1
C1—C2—H2A	121.0	C17—C16—C15	110.0 (2)
C2—C3—C4	118.5 (2)	C17—C16—H16A	109.7
C2—C3—C12	119.6 (2)	C15—C16—H16A	109.7
C4—C3—C12	121.8 (2)	C17—C16—H16B	109.7
C3—C4—C5	123.5 (2)	C15—C16—H16B	109.7
C3—C4—C9	118.3 (2)	H16A—C16—H16B	108.2
C5—C4—C9	118.2 (2)	C16—C17—C13	111.5 (2)
C6—C5—C4	120.8 (2)	C16—C17—H17A	109.3
C6—C5—H5	119.6	C13—C17—H17A	109.3
C4—C5—H5	119.6	C16—C17—H17B	109.3
C5—C6—C7	120.8 (2)	C13—C17—H17B	109.3
C5—C6—H6A	119.6	H17A—C17—H17B	108.0
C7—C6—H6A	119.6	O3—C18—O2	123.9 (2)
C8—C7—C6	120.3 (2)	O3—C18—C19	117.8 (2)
C8—C7—H7	119.9	O2—C18—C19	118.3 (2)
C6—C7—H7	119.9	C24—C19—C20	120.2 (2)
C7—C8—C9	120.2 (2)	C24—C19—C18	119.9 (2)
C7—C8—C11	119.6 (2)	C20—C19—C18	119.8 (2)
C9—C8—C11	120.1 (2)	C21—C20—C19	117.0 (2)
N1—C9—C8	118.1 (2)	C21—C20—H20	121.5
N1—C9—C4	122.2 (2)	C19—C20—H20	121.5
C8—C9—C4	119.6 (2)	C22—C21—C20	124.0 (2)
F3—C10—F2	108.0 (2)	C22—C21—N3	117.7 (2)
F3—C10—F1	106.2 (2)	C20—C21—N3	118.2 (2)
F2—C10—F1	105.0 (2)	C21—C22—C23	119.0 (2)
F3—C10—C1	112.7 (2)	C21—C22—H22A	120.5
F2—C10—C1	112.3 (2)	C23—C22—H22A	120.5
F1—C10—C1	112.1 (2)	N4—C23—C24	120.7 (2)
F5—C11—F6	106.4 (2)	N4—C23—C22	121.4 (2)
F5—C11—F4	106.9 (2)	C24—C23—C22	117.8 (2)
F6—C11—F4	105.7 (2)	C19—C24—C23	121.9 (2)
F5—C11—C8	113.6 (2)	C19—C24—H24	119.1
F6—C11—C8	112.1 (2)	C23—C24—H24	119.1
F4—C11—C8	111.6 (2)		
			
C9—N1—C1—C2	3.1 (4)	C9—C8—C11—F4	61.3 (3)
C9—N1—C1—C10	−178.5 (2)	C2—C3—C12—O1	18.0 (3)
N1—C1—C2—C3	−2.3 (4)	C4—C3—C12—O1	−164.9 (2)
C10—C1—C2—C3	179.3 (2)	C2—C3—C12—C13	−102.9 (3)
C1—C2—C3—C4	−0.9 (3)	C4—C3—C12—C13	74.2 (3)
C1—C2—C3—C12	176.3 (2)	C14—N2—C13—C17	−55.4 (3)
C2—C3—C4—C5	−177.6 (2)	C14—N2—C13—C12	179.6 (2)
C12—C3—C4—C5	5.3 (4)	O1—C12—C13—N2	66.0 (2)
C2—C3—C4—C9	3.0 (3)	C3—C12—C13—N2	−171.6 (2)
C12—C3—C4—C9	−174.1 (2)	O1—C12—C13—C17	−55.4 (3)
C3—C4—C5—C6	−177.9 (2)	C3—C12—C13—C17	67.0 (3)
C9—C4—C5—C6	1.4 (4)	C13—N2—C14—C15	55.0 (3)
C4—C5—C6—C7	0.7 (4)	N2—C14—C15—C16	−54.2 (3)
C5—C6—C7—C8	−1.0 (4)	C14—C15—C16—C17	56.0 (3)
C6—C7—C8—C9	−0.9 (4)	C15—C16—C17—C13	−57.8 (3)
C6—C7—C8—C11	177.1 (2)	N2—C13—C17—C16	56.6 (3)
C1—N1—C9—C8	−179.1 (2)	C12—C13—C17—C16	176.7 (2)
C1—N1—C9—C4	−0.6 (3)	O3—C18—C19—C24	−178.8 (2)
C7—C8—C9—N1	−178.4 (2)	O2—C18—C19—C24	1.1 (3)
C11—C8—C9—N1	3.6 (3)	O3—C18—C19—C20	−0.2 (3)
C7—C8—C9—C4	3.0 (4)	O2—C18—C19—C20	179.7 (2)
C11—C8—C9—C4	−175.0 (2)	C24—C19—C20—C21	0.9 (3)
C3—C4—C9—N1	−2.3 (3)	C18—C19—C20—C21	−177.7 (2)
C5—C4—C9—N1	178.3 (2)	C19—C20—C21—C22	−0.8 (4)
C3—C4—C9—C8	176.1 (2)	C19—C20—C21—N3	178.9 (2)
C5—C4—C9—C8	−3.2 (3)	O4—N3—C21—C22	−176.4 (2)
N1—C1—C10—F3	170.9 (2)	O5—N3—C21—C22	4.3 (3)
C2—C1—C10—F3	−10.5 (3)	O4—N3—C21—C20	3.9 (3)
N1—C1—C10—F2	−66.8 (3)	O5—N3—C21—C20	−175.4 (2)
C2—C1—C10—F2	111.7 (3)	C20—C21—C22—C23	0.0 (4)
N1—C1—C10—F1	51.2 (3)	N3—C21—C22—C23	−179.6 (2)
C2—C1—C10—F1	−130.3 (2)	C21—C22—C23—N4	−177.1 (3)
C7—C8—C11—F5	122.3 (3)	C21—C22—C23—C24	0.6 (4)
C9—C8—C11—F5	−59.7 (3)	C20—C19—C24—C23	−0.3 (4)
C7—C8—C11—F6	1.7 (3)	C18—C19—C24—C23	178.3 (2)
C9—C8—C11—F6	179.7 (2)	N4—C23—C24—C19	177.3 (3)
C7—C8—C11—F4	−116.7 (3)	C22—C23—C24—C19	−0.4 (4)

### Hydrogen-bond geometry (Å, °)

**Table:**

*D*—H···*A*	*D*—H	H···*A*	*D*···*A*	*D*—H···*A*
O1—H1···O2	0.84 (1)	1.83 (1)	2.669 (3)	178 (3)
N4—H41···O2^i^	0.88 (1)	2.04 (1)	2.900 (3)	164 (3)
N2—H21···O3^ii^	0.89 (1)	1.87 (1)	2.717 (3)	159 (3)
N4—H42···O5^iii^	0.88 (1)	2.27 (2)	3.101 (3)	159 (3)
N2—H22···O2W	0.89 (1)	2.05 (1)	2.916 (4)	165 (3)
N2—H22···O3W	0.89 (1)	1.98 (2)	2.727 (5)	141 (3)
OWw—H1W1···O1	0.84 (1)	2.08 (4)	2.867 (5)	156 (8)
O1W—H1W2···F6^iv^	0.84 (1)	2.47 (7)	2.867 (5)	110 (6)
O2W—H2W1···O1W	0.85 (1)	2.07 (2)	2.859 (6)	154 (4)
O2W—H2W2···O4^v^	0.85 (1)	2.30 (2)	3.119 (4)	165 (4)
O3W—H3W1···O3	0.84 (1)	2.21 (1)	3.049 (5)	174 (7)
O3W—H3W2···O4^v^	0.84 (1)	2.37 (3)	3.158 (5)	156 (7)
O3W—H3W2···O5^v^	0.84 (1)	2.32 (6)	3.016 (5)	140 (7)

Symmetry codes: (i) −*x*+1, −*y*+2, −*z*+1; (ii) −*x*, −*y*+2, −*z*+1; (iii) −*x*+1, −*y*+3, −*z*+1; (iv) *x*, *y*+1, *z*; (v) −*x*, −*y*+3, −*z*+1.
